# Editorial: Perception recovery and augmentation in medical robotics

**DOI:** 10.3389/fnbot.2022.1058793

**Published:** 2022-11-02

**Authors:** Wenfeng Zheng, Jian Huang, Chao Liu, Bo Yang

**Affiliations:** ^1^School of Automation Engineering, University of Electronic Science and Technology of China, Chengdu, China; ^2^School of Artificial Intelligence and Automation, Huazhong University of Science and Technology, Wuhan, China; ^3^LIRMM, UMR5506, University of Montpellier - CNRS, Montpellier, France

**Keywords:** perception recovery, perception augmentation, teleoperation, human-machine interactive, medical robotics

Artificial intelligence technology has been applied to complex problems in many fields. From the perspective of neuroscience, the continuous development of neuroscience can explain the deep mechanism of human brain work, thus helping researchers develop more powerful artificial intelligence algorithms. From the perspective of brain computer interface, in-depth research in this field can generate more powerful intelligence through the interaction between human brain and machine. Empowered by the recent advances in artificial intelligence and robot technology, medical robotics have found wider and deeper applications in the health care practice. Except helping the doctors in diagnosis and medical treatment, medical robotic systems also contribute to the development of medical informatization while effectively alleviating the shortage of medical resources. On the other hand, it is noticed that, with the use of computer technology and novel robotic tools, doctors may lose some of their intuitive human perception during the robot-aided diagnoses/operations. In this case, remedial and even better solutions have been proposed to address this issue in medical robotics. Examples include high definition endoscope to provide the surgeon with *in vivo* visual perception of wider working environment, force and tactile feedback generated by the joystick of the console to provide the surgeon with accurate and scaled tactile perception, etc. These solutions can replace and even augment some human perception capabilities with machine perception. It is believed that the advanced machine perception will greatly contribute to the benefits of both the doctors and patients and therefore to the overall progress of medical robotics.

After a careful peer-review process, this editorial presents the manuscripts selected for publication in the Research Topic “*Perception recovery and augmentation in medical robotics*” of *Frontiers in Neurorobotics*, which includes seven articles. These articles are original research papers covering multiple aspects of medical robotics that include individualized gait guidance in rehabilitation walking training, cone-beam computerized tomography, endoscopic imaging, robust classification of natural hand grasp type based on electromyography, assistant robots and time-delay force feedback teleoperation system.

In the article “*Effects of individualized gait rehabilitation robotics for gait training on hemiplegic patients: Before-after study in the same person*” (Guo et al.), the authors aimed to explore the effect of individual gaits on energy consumption situations during gait rehabilitation training for hemiplegic patients with lower-limb exoskeleton robots. A total of 9 unilateral-hemiplegic patients were recruited for a 2-day experiment. The results show that using individualized gait guidance in rehabilitation walking training can significantly improve energy efficiency for hemiplegic patients with stroke.

In the paper “*Sparse angle CBCT reconstruction based on guided image filtering*” (Xu et al.), the authors propose a sparse angle Cone-beam Computerized Tomography (CBCT) reconstruction algorithm based on Guided Image Filtering, which combines the classic Simultaneous Algebra Reconstruction Technique and the Total p-Variation minimization. The proposed method can suppress noise and artifacts while preserving edge and texture information in reconstructed images.

In the paper “*Improved feature point pair purification algorithm based on SIFT during endoscope image stitching*” (Liu et al.), an improved pair purification algorithm based on Scale invariant feature transform (SIFT) is proposed to solve the problem that there are many matching errors because there are many similar regions in endoscopic images, which will affect the final stitching effect. The experimental results show that the matching rate of feature points by using the improved feature pair purification algorithm is greatly improved.

The authors of the article “*Improving the robustness of human-machine interactive control for myoelectric prosthetic hand during arm position changing*” (Ke et al.) provided a framework for robust hand grasp type classification during dynamic arm position changes, improving both the “hardware” and “algorithm” components. In the hardware aspect, co-located synchronous EMG and force myography (FMG) signals are adopted as the multi-modal strategy. In the algorithm aspect, a sequential decision algorithm is proposed by combining the RNN-based deep learning model with a knowledge-based post-processing model. The average classification accuracy can be improved compared with other traditional methods or DL methods.

In the article “*Simultaneous localization and mapping algorithm based on the asynchronous fusion of laser and vision sensors*” (Xing et al.), a simultaneous localization and mapping algorithm based on the weighted asynchronous fusion of laser and vision sensors is proposed for an assistant robot. This method can effectively use the redundant data in the vision sensor and improve the tracking accuracy of the algorithm. Compared with the synchronous fusion method, the asynchronous fusion algorithm has a more accurate prior, faster operation speed, higher pose estimation frequency, and more accurate positioning accuracy.

In the article “*Control of time delay force feedback teleoperation system with finite time convergence*” (Wang et al.), by combining the terminal sliding mode control method with the neural network adaptive control method, a bilateral continuous finite time adaptive terminal sliding mode control method is designed for the combined teleoperation system. The effectiveness of the proposed control scheme is verified by MATLAB Simulink numerical simulation, and the numerical analysis of the results shows that the method has better system performance.

In “*Adaptive control of time delay teleoperation system with uncertain dynamics*” (Lu et al.), a bilateral adaptive control method based on position error-based structure (PEB) control structure is designed for a class of time-delay force feedback teleoperation system without external interference and internal friction to study the uncertainty of dynamic parameters and time delay. The controller designed in this paper is successfully applied to the teleoperation system composed of a two-degree of freedom rotating manipulator as the master robot and the slave robot.

We would like to thank all the authors and reviewers for their contributions to the Research Topic. We hope that this SI and the seven published articles will help the interested readers to better understand perception recovery and augmentation in medical robotics and that they will help inspire further research and new results within the targeted domain.

## Author contributions

JH, CL, and BY helped refine and review the manuscripts after WZ prepared them with contributions from all co-authors. All authors contributed to the article and approved the submitted version.

## Conflict of interest

The authors declare that the research was conducted in the absence of any commercial or financial relationships that could be construed as a potential conflict of interest.

## Publisher's note

All claims expressed in this article are solely those of the authors and do not necessarily represent those of their affiliated organizations, or those of the publisher, the editors and the reviewers. Any product that may be evaluated in this article, or claim that may be made by its manufacturer, is not guaranteed or endorsed by the publisher.

